# Paradox lost on the U.S.-Mexico border: U.S. Latinas and cesarean rates

**DOI:** 10.1186/s12884-018-1701-9

**Published:** 2018-04-03

**Authors:** Theresa Morris, Amanda Gomez, Miriam Naiman-Sessions, Christine H. Morton

**Affiliations:** 1 Department of Sociology, Texas A&M University, MS4351, College Station, TX 77843 USA; 2 Independent Scholar, Bryan, TX USA; 3 Independent Scholar, Helena, MT USA; 4 Independent Scholar, Redwood City, CA USA

**Keywords:** Cesarean, Childbirth, Hispanic health paradox, U.S-Mexico border, Listening to Mothers III, Intersectional theory

## Abstract

**Background:**

We apply Intersectional Theory to examine how compounded disadvantage affects the odds of women having a cesarean in U.S.-Mexico border hospitals and in non-border hospitals. We define U.S. Latinas with compounded disadvantage as those who have neither a college education nor private health insurance.

**Results:**

Analyzing quantitative and qualitative data from Childbirth Connection’s Listening to Mothers III Survey, we find that, consistent with the notion of the Latinx Health Paradox, compounded disadvantage serves as a protective buffer and decreases the odds of cesarean among women in non-border hospitals. However, the Latinx Health Paradox is absent on the border.

**Conclusion:**

Our data show that women with compounded disadvantage who give birth on the border have significantly *higher* odds of a cesarean compared to women without such disadvantage. Further, women with compounded disadvantage who give birth in border hospitals report receiving insufficient prenatal, pregnancy, and postpartum information, providing a direction for future research to explain the border disparity in cesareans.

## Background

The U.S.-Mexico border region is a politically significant geographical region, as evidenced by its centrality in the 2016 United States presidential campaign. In fact, this strip of land, which spans 2000 miles, 48 counties, and four states, is often used to highlight hot-button political issues like immigration, crime, and poverty [1]. The region is ethnically, culturally, and economically distinct from the rest of the U.S. population. For instance, in 2000, Latinos and Latinas comprised 49% of the population in border counties—a far greater percentage than the 12.6% of Latinos in the general population in 2000 [2]^1^. Further, compared to the overall U.S. population, individuals in the border region have a lower income per capita. This income disparity is greater for Latinos and Latinas in the border region, who make less than both whites in the border region and Latinos and Latinas in non-border regions. Border residents also have a lower level of educational attainment than the general population and are less likely to have private health insurance, suggesting the importance of looking at compounded disadvantage, especially in the border region [2].

However, despite Latinos’ and Latinas’ lower socioeconomic status, Latinx ethnicity in the United States is often correlated with positive health outcomes – a phenomenon referred to as the Hispanic or Latinx Health Paradox [3]^2^. This phenomenon has been applied to maternal-fetal health, although findings typically focus on fetal rather than maternal outcomes. For example, studies have found that Latinas have lower pre-term birth, low-birth weight, and maternal mortality rates compared to non-Latinas in the U.S. [4–9].

Thinking about the border and the Latinx Health paradox led us to wonder how this geographic and socioeconomic context might affect maternal outcomes on the border, specifically women’s odds of having a cesarean, an important maternal outcome because cesareans carry greater health risks than vaginal birth [10]. Thus, in this study we examine how compounded disadvantage—defined as being Latina without private health insurance or a college degree—affects the odds of cesarean in border and in non-border hospitals. Does the Latinx Paradox protect Latinas, especially Latinas with compounded disadvantage, from cesareans? If it does, is this paradox present in border and in non-border hospitals?

The World Health Organization concludes that “[a]t the population level, caesarean section rates higher than 10% are not associated with reductions in maternal and newborn mortality rates” [11]. U.S. Healthy People 2020 sets a target for a low-risk, first birth cesarean rate of 23.9% [12]. Despite these goals and statements, the cesarean rate has increased in both developing and developed nations and for all groups of women [13]. As of 2016, the United States’ cesarean rate was 31.9% [14]. This rate varies greatly among women, hospitals, and states. For example, certain individual health conditions, prior cesarean, hospital characteristics, and state have all been shown to affect a woman’s chance of having a cesarean [15, 16]. The likelihood of cesarean is also affected by social factors like race, ethnicity, and socioeconomic status [15, 17]. African American women have a higher risk-adjusted rate of cesarean than white women; but the data are inconsistent for Hispanic and Pacific Islander women, with studies indicating equivalent, lower, and higher rates than white women [18–21]^3^. The few studies that have analyzed educational attainment—a proxy measure of socioeconomic status—and its effect on cesarean rates have provided mixed results, but the majority of national and state studies find that the probability of having a cesarean increases with level of maternal educational attainment [22–24], although some studies show just the opposite effect [25]^4^. Further, women with private insurance have higher rates of cesareans compared to those with Medicaid and those who are uninsured [15, 26–28].

A few studies have analyzed cesarean rates on the U.S-Mexico border. An analysis of birth certificate data compared cesarean rates in Mexico to the cesarean rate of Hispanics in the United States and residents in border regions (both south and north of the border). This study found that U.S. border residents have a higher cesarean rate than the U.S. Hispanic cesarean rate. Both of these groups have a lower cesarean rate than the Mexican rate of 44.5% (in 2009). Additionally, a Texas-based study found that border residents, particularly Hispanic women, have a higher rate of cesareans than non-border residents [29]. Although these studies shed light on border-region cesarean rates, they neither explore the reason for the difference in border rates nor integrate women’s voices into their analysis.

Our study fills a gap in this literature in two ways. First, we focus on how compounded disadvantage affects the odds of Latinas having a cesarean in border and non-border hospitals, which allows us to add nuance to the application of the Latinx Health Paradox on maternal outcomes. Second, we include an analysis of qualitative data on women’s birth experiences to better explore whether the Latinx paradox with regard to cesarean rates persists on the border.

## Methods

We used data from Childbirth Connection’s Listening to Mothers III survey: a nationally representative survey of 2400 women who gave birth in U.S. hospitals in 2011 and 2012 [30]. This was an online survey of women aged 18–45 who had given birth between July 1, 2011 and June 30, 2012 and who could complete the survey in English. Participants took the survey between October and December 2012. Women who completed the survey were re-contacted and invited to complete a follow-up survey between January 29, 2013 and April 15, 2013. Almost half of the women (1072 or 45%) completed this follow-up survey, which included open-ended questions about their pregnancy and birth experiences and questions asking in what hospital, city, and state they gave birth. We analyzed data from this follow-up survey because it includes the geographic location of the women’s birth. Although the post-partum dataset is weighted to make the data representative of the national population of English-speaking women aged 18–45 who gave birth to a singleton baby in hospitals in 2011 and 2012, we do not use the weight due to deletion of 138 women from the dataset because they did not provide the hospital, city, and/or state of the birth. For the quantitative analysis, we drop cases list-wise to allow for the most data to be used in each analysis. In order to provide a greater number and variety of experiences to analyze for the qualitative data, we analyze responses from women who provided information for hospital location and ethnicity, even if they did not provide information for educational attainment and/or insurance status.

The dependent variable is mode of delivery, with attributes of cesarean or vaginal birth. The independent variable in this study is compounded disadvantage, measured as being Latina, without a college degree and without private insurance. We also ran analyses that examine the independent effect of ethnicity (Latina or non-Latina), educational attainment (four-year college degree and above or less than four-year college degree), and private health insurance status (absent or present). We further integrated border region into our analysis. We used the Centers for Disease Control and Prevention’s definition of the border, which is based on the La Paz Agreement and defined as “the area situated 100 kilometers on either side of the inland and maritime boundaries between [the United States and Mexico]” [1]. Our study focuses on the United States border region (i.e. 100 km north of the U.S. Mexico border). In order to contextualize the study, we included descriptive measures of average parity and age, as well as the percent of births involving pre-term infants (less than 37 weeks’ gestation), term infants (37–42 weeks’ gestation), and post-term infants (42 weeks’ or more gestation), and percent of cesareans for malpresentation, the closest measure in the dataset to indicate non-vertex presentation.

The qualitative measures of birth experiences are derived from two questions in the post-partum survey: 1) If you have a baby in the future, would you want to give birth again in the same hospital? Why or why not? 2) If you could go back in time and give yourself any advice or information as you were going into your birth, what would it be?

We analyzed the data in two ways: quantitatively and qualitatively. For the quantitative analysis, we used multiple logistic regression models to examine the association between individual characteristics in the full sample (Model 1), compared to how those same individual characteristics interact to create a compounded disadvantage in the non-border (Model 2) and border (Model 3) subsamples. Compounded disadvantage is measured using an interaction term (coded as being Latina with no private insurance and no college education). Results are presented as odds ratios (i.e. exponentiated coefficients). We used SPSS version 24.0 to analyze the quantitative data.

For the qualitative analysis, due to relatively short responses, we coded overall answers as positive or not positive. The responses coded as ‘not positive’ included those that were neutral-to-negative, as they were a stark contrast to the glowing responses provided by happy, satisfied women. After coding overall responses as positive or not positive, we examined the themes that emerged from the data related to birth experiences. Women did not provide their names in the survey. Thus, all names used in the analysis are fictitious. We used Dedoose Version 7.5.10 (SocioCultural Research Consultants, LLC, Manhattan Beach, California), a mixed-methods software program, to code and analyze the qualitative data.

## Results

### Quantitative results

#### Univariate and Bivariate analysis

Table 1 contains descriptive statistics from our sample, which we separate by whether women gave birth in hospitals located on the U.S.-Mexico border or not on the border. While Latinas are greatly outnumbered by non-Latinas in the non-border region (14.9% Latinas), they only slightly out number Latinas in the border region (45.1% Latinas). A little more than half (51.5%) of the women had a college degree in the non-border region while fewer than half (46.2%) of the women in the border region had a college degree. Sixty-nine percent of women in the non-border region had private health insurance compared to 56.9% of women in the border region. These results are expected based on previous demographic studies conducted on the border region. Historically, the border region has been an area with low educational attainment and income per capita (a measure of socioeconomic status) [2]. As evidence, 26% of the women in the border region fell into the compounded disadvantaged group, while only 6% of non-border women fell into this group. We also include indictors of parity (1.9 for both groups); mean maternal age (28.4 for the border group and 30.6 for the non-border group); gestational age (Nearly all women delivered at term in both groups with 7.7% and 8% having pre-term births and 0% and 1% having post-term births in the border and non-border groups, respectively); previous cesarean (25% for the border group and 19% for the non-border group); and cesarean for malpresentation (0% for the border group and 2.4% for the non-border group).

**Table 1:** Descriptive statistics by region

	Border Region	Non-Border Region
Ethnicity		
Latina	45.1%	14.9%
Health Insurance Status		
Private	56.9%	69.3%
Education		
College Degree or more	46.2%	51.5%
Compounded Disadvantage	25.5%	6.4%
Cesarean Rate	32.7%	28.7%
Gestational Age		
Pre-term (< 37 weeks)	7.7%	8.0%
Term (37–41 weeks)	92.3%	91.0%
Post-term (42 or more weeks)	0.0%	1.0%
Mean Parity	1.9	1.9
Mean Maternal Age	28.4	30.6
Previous Cesarean	25%	19%
Cesarean for Malpresentation	0%	2.4%
	*N* = 52	*N* = 941

**Table 2:** Odds Ratios for logistic regression analysis of cesarean section delivery

	Model 1	Model 2	Model 3
	Entire Sample	Non-Border Sample	Border Sample
Latina	.920		
Private Health Insurance	1.162		
College Degree	1.133		
Border	1.194		
Compounded Disadvantage		.532*	3.759*
Constant	.354**	.422**	.310**
	*N* = 960	*N* = 933	*N* = 51

(* *p* ≤ 0.05; **p ≤ 0.01; one-tailed test)

**Fig. 1:**
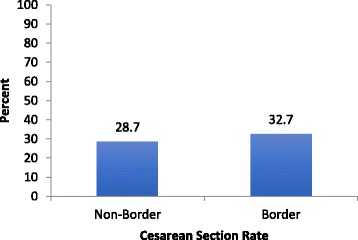
Cesarean Section Rate by Region, LTMIII, 2011–2012. Cesarean Section Rate

Figure 1 examines whether region affects the cesarean rate. Cesarean rates for the non-border region and border region were 28.7% and 32.7% respectively, a non-significant difference. According to CDC 2012 birth data, the national cesarean section rate was 33% in 2012 [31].

### Multivariate analysis

Table 2 contains the multivariate analysis. First, we examined the effect of women’s individual characteristics on the cesarean rate for the entire sample (Model 1). We find that none of these characteristics individually are significantly related to the cesarean rate. This is not surprising as cesarean rates increased for women of all races and ethnicities until 2009; although recently rates of low-risk cesareans have declined slightly among non-Hispanic white, non-Hispanic black, and Hispanic women [8]. Then, we separated the data into border and non-border subsamples by hospital location where women gave birth and used an interaction term to analyze the effect of compounded disadvantage on the odds of cesarean section. Through these analyses, we find that compounded disadvantage is statistically significant and negative for the non-border sample (Model 2)—compounded disadvantage (i.e. being Latina with no private insurance and no college education) decreases the odds of a cesarean section by 47%—and statistically significant and positive for the border sample (Model 3)—compounded disadvantage increases the odds of cesarean by 276%. In regard to cesarean rates, the Latinx Paradox is lost along the border. That is to say, Latinas with public health insurance and low educational attainment—compounded disadvantage—are more likely to have cesareans in the border region, while women with these same characteristics are less likely to have a cesarean in the non-border region.

### Qualitative results

We analyzed the qualitative data to contextualize the quantitative findings by analyzing the experience of (1) Non-Latinas in the border region; (2) Latinas in the border region; (3) Non-Latinas in the non-border region; and (4) Latinas in the non-border region. Within each of these groups, we focus upon themes we see in the compounded advantaged or compounded disadvantaged groups. Recall that for the qualitative analysis, we focused on the experiences of the group with compounded advantaged (non-Latinas with private health insurance and a college degree) compared to the group with compounded disadvantage (Latinas without private health insurance or college degree).

#### Birth experiences for non-Latinas in the border region

##### Overall response

The vast majority of non-Latina women on the border (twenty out of twenty-eight) were positive about their births while six provided either neutral or non-responses and two were negative. Michelle, a college-educated mother with private health insurance and in her late thirties, reflected a commonly shared sentiment among non-Latina women on the border: “Yes, I love the hospital and staff. They are so attentive and friendly. They give you the sensation that you are the only person in the hospital [with] needs and that my birth is special and important.” The majority of those who responded positively about their births were non-Latinas in the advantaged group on the border who had given birth vaginally (eleven out of twenty). Out of the three non-Latinas in the disadvantaged group on the border, two had vaginal births and gave positive responses. The remaining woman, Kristin, had a cesarean and did not give a positive response. When asked whether she would return to the same hospital, she responded: “I have no choice which hospital I give birth at. Our military medical makes us go to the [hospital] if we can make it. And, with c-sections with both living children, my third will most likely be a repeat c-section.” While Kristin’s answer is not inherently negative, it does reflect constrained hospital choice and a resignation with her history of repeat cesarean.

##### Reactions to cesarean

All five women who spoke about cesareans indicated that they either wished they had not had a cesarean in the past or that they did not want a cesarean in the future. For instance, Iliana, a college educated mother with private health insurance in her late twenties, simply stated that if she could go back in time and give herself advice, she “would have attempted a natural birth instead of a 2^nd^ c-section.” Another mother, also in the advantaged group, took her cesarean history and her hospital’s vaginal birth after cesarean (VBAC) rate into consideration when deciding whether she would return to her hospital for future births: “I might. I want to do a vbac and a midwife center is the best place to do one. The hospital I went to is not known for its great vbac success rate. I am afraid I would end up with another c-section if I went back to the same hospital.” Two women equated their cesareans with a loss of efficacy. Sharing a similar sentiment to Kristin’s response above, Melanie, a privately insured mother with an associate’s degree, did not have any advice for past self: “I don’t think there is anything I would tell myself. I had a c-section and did not push or anything, so I doubt anything I would tell myself would have an effect.”

##### Remarks about staff

While most non-Latinas were happy with the staff attending their birth, one woman had a surprising comment about the staff at her hospital. When asked if she would return to this facility, Jessica, a college educated mother on government insurance, said, “No. The staff spoke very broken English and seemed to get upset with me when I didn’t understand them.”

#### Birth experiences for Latinas in the border region

##### Overall response

Similar to non-Latina women on the border, most Latinas on the border (nineteen out of twenty-three) had positive birth experiences. Cassandra, a teenage mother insured through Medicaid, gave a typical response: “I would give birth to my next child at the same hospital. The staff were so great with me. I enjoyed their presence in the room. I know many say hospital food is the worst but I loved it. I miss hospital food the most.” Like Cassandra, Latinas on the border in the disadvantaged group who had vaginal births had the highest number of positive responses (six out of nineteen). Latinas on the border in the disadvantaged group who had cesareans, however, had the highest number of non-positive responses (three out of four). Further, all four border Latinas who had responses that were non-positive had cesareans. Two women directly stated that they would not return to the hospital, one woman noted that she had a tubal ligation and thus would not be having children again (no comment about hospital experience), and one woman did not give a clear answer about returning to her hospital. All Latinas on the border who had vaginal births gave positive responses.

##### Tubal ligation

While a few non-Latinas on the border mentioned that they were “not planning” on having any more children, none of them directly referred to tubal ligations. Two Latinas on the border who had cesareans, however, directly commented on having this procedure. Both of these mothers were in the disadvantaged group. Another border Latina, a mother in her early to mid-twenties without a college degree, wanted information about when she could get a tubal ligation.

##### Requesting information

While no non-Latinas on the border indicated that they wanted more information from their healthcare providers or other resources, two Latinas on the border expressed a desire for more information during pregnancy, birth, and postpartum. Laura, a high school educated mother insured through Medicaid, mentioned communication with staff more generally: “i would ask nurses when im unsure.” Ida, a mother in her early to mid-twenties without a college degree, had a list of specific topics she wanted information about: “How many C-sections can you have at a certain age, information on preeclampsia, exercise tips after having a c-section, when can I get my tubes tied, etc.” Both Laura and Ida had cesareans. Briana, a mother without a college degree and insured through Medicaid, provided the most negative response: “The doctor’s attentiveness could have been a little better, and they didn’t have to wait so long. If a patient says that they don’t feel well, it is because they don’t feel well – not because they want to be stupid and wait for hours in the waiting room”^5^. Brianna had a cesarean.

#### Birth experiences for Latinas in the non-border region

##### Overall response

The majority of Latinas in non-border regions (79.1%) provided positive comments about their births. Nina, a mother with a high school diploma and Medicaid, gave a characteristic response: “yes i would love too give birth in the same place because i love how the doctors and nurses were with me. they were so nice and respectful and helpful. cause being a first time mommy is kinda scare you dont know whats going to happen in the labor room but they sure cheered me up. god bless them.” Out of all non-border Latinas, women in the disadvantaged group who had vaginal births had the highest percentage of positive responses (35.8%). Interestingly, this same group of women had the highest percentage of neutral to negative responses (25.0%). Although some women cited reasons that they would not return to their hospital for another birth (dissatisfaction with staff, rooms, facility), the majority of them did not give a specific reason. Examples of responses coded as non-positive (neutral to negative) are “I don’t know,” “no,” and “maybe.” However, one of these non-positive responses was from Kayla, a mother with her associate’s degree and insured through Medicaid, who stated, “The hospital i went to was in the poor part of town. i would like to go where the rich go, downtown [city].”

##### Not having any more babies

Similar to the border non-Latinas, nine non-border Latinas stated that they were not planning on having any more children. When asked if she would return to her hospital, Rosa, a mother in her forties with a high school diploma and private insurance, responded, “I can’t have anymore children but if I could I would use the same hospital. They took excellent care of me and my baby.” Like Rosa, many women said they “can’t” or “won’t” be giving birth again without providing further explanation. We cannot say whether or not these women had a tubal ligation performed, but it is interesting that, in contrast, every border Latina explained that they were not having more babies specifically because they had a tubal ligation.

##### Reactions to cesarean

Only two non-border Latinas mentioned cesareans at all in their responses. And unlike border Latinas, non-border Latinas did not give their past selves advice about avoiding the procedure. Rather, both women brought up their cesareans to highlight interactions with hospital staff. For instance, Lidia, a mother with an associate’s degree and Medicaid, said that she loved her hospital because “they informed me of the reasons why they recommended certain things, including the c-section.” Karla, a mother with an associate’s degree and private insurance, said she would not go back to her hospital partly because she had to “deal with a c-section all by [herself]” and because her “dr didn’t discharge [her] or visit [her] after [her] c section.” However, both of these women appear to have understood that cesareans are serious procedures. This explains why Lidia appreciated her hospital staff’s thorough information about the need for surgery and why Karla was dissatisfied with her doctor’s absence after the procedure.

##### Information

Five non-border Latina women spoke directly about issues regarding information. Two of these responses were negative, two were positive, and one was neutral. Similar to Lidia’s experience above, a woman in her early twenties with Medicaid said that she would return to her hospital because she was “given all the information.” Conversely, Olivia, a mother who completed some college and has Medicaid, gave herself the following advice: “Fight like the Mama bear you are. Do not let anyone tell you how to parent your child, especially doctors that try to bully you by giving misleading information. You can do it, because you have maternal instincts that will overcome so much in the future.” When asked whether she would return to her hospital, another woman who provided a negative response said she would not because she felt “rushed” and did not know who to ask for help. Both of these negative responses are not simply about information. Lack of information (and misleading information) resulted in these women feeling dissatisfied with their health care providers and hospital. The non-border Latina who provided a neutral response said that she wanted to take a natural birth class.

## Discussion

Intersectional Theory posits that social identities, such as race, class, and gender, interact simultaneously to create multidimensional systems of oppression and lead to different experiences and outcomes [32]. That is, social disadvantage can have a multiplicative effect, as individuals with social identities belonging to multiple oppressed groups experience compounding social disadvantage. In terms of maternal health, our data show that women’s individual characteristics of ethnicity and SES do not independently affect their chance of cesarean. However, we find that the likelihood of cesareans is influenced by intersecting identities in particular geographic contexts. In the non-border region, Latinas with low levels of education and public health insurance have a lower rate of cesareans than non-Latinas or Latinas with high education levels and private insurance. However, in border regions, Latinas with compounded disadvantage (low education/no private health insurance, have a higher cesarean rate than other women. This finding provides validity for applying Intersectional Theory to explain mode of delivery in certain contexts, since most studies tend to look at these factors individually. In this study, we find that the Latinx Health Paradox for cesarean outcome is lost on the border.

A strength of our study lies in our use of Intersectional Theory, which informed our creation of a “compounded disadvantage” variable. Using this variable, our analysis shows that intersecting statuses affect cesarean rates in particular geographic contexts for particular women. Research on variations in cesarean rates have found differences by hospital and geographic regions [15] that cannot be fully explained by medical or pregnancy characteristics of women. Our analysis goes further to demonstrate that Latinas who are multiply disadvantaged in terms of education and health-insurance status have greater risks of cesarean if they give birth in border hospitals. Future studies that examine drivers for disparities in mode of birth could apply similar methods and theories to better understand reasons for variation in or overuse of cesareans.

There are limitations to our study that should be considered in analyzing the results. First, we are limited to the LTMIII methodology, which required women to take the survey in English, a major consideration for a study focused on the border region where some women may be primarily Spanish speakers. New data from the 2017 Listening to Mothers California may solve this problem by oversampling Latinas and also by fielding the survey in both English and Spanish, though only in one state. While we cannot generalize our study to the U.S. population as a whole, the application of Intersectional Theory to investigate disparities in mode of birth provides an innovative way to explore non-medical factors that contribute to an overuse of cesareans. Second, the survey from which we draw our data has the limitation of selection bias, which is very difficult to control. However, all surveys, particularly those in which participation is voluntary, share this problem. In short, some women are more inclined than others to respond. However, the data we use are the only data from a representative sample of U.S. women who gave birth that also include the geographic location in which they gave birth.

## Conclusions

These findings deserve further exploration. First, it is important for clinicians working in the border region to understand the increased risk for disadvantaged Latinas to have cesareans. Hospital leaders already working to reduce cesarean rates may want to analyze their institutional rates by using these intersecting statuses to understand the processes and policies that produce these outcomes. Our qualitative analysis shows that Latinas who give birth in border hospitals desire more information throughout pregnancy and childbirth. Interestingly, this desire for information is less visible among non-Latinas who gave birth in a border hospital and among Latinas who gave birth in a non-border hospital, perhaps reflecting their lower rates of cesarean overall. Thus, clinicians and birth professionals should make an extra effort to get relevant information about childbirth and cesarean prevention to Latinas on the border. Second, Latinas on the border were the only group of women in our analysis to discuss tubal ligation. Because of the history of forced sterilization of Latinas in the United States, it is concerning to find a pattern of Latinas on the border discussing sterilization, whereas this topic is absent from the responses of other groups of women [33]. We do not know the circumstances of these sterilizations. It is beyond our data to determine whether sterilizations were coerced or chosen because women understand that they can be done easily during a cesarean or for some other reason. However, again, hospitals should be cautious of how sterilization is discussed with patients and make sure that the discussion is culturally sensitive.

## Abbreviations

LTMIII: Listening to Mothers III
VBAC: Vaginal Birth After Cesarean

## Footnotes

1 The 2000 Census includes the most recent survey of the U.S. southern border. The 2010 Census Brief on the Hispanic population does not report the ethnic composition of the border region.

2 Although this phenomenon is commonly referred to as the “Hispanic Paradox,” we choose to use the term “Latinx Paradox” because our study centers on women from Latina America.

3 The Centers for Disease Control and Prevention does not provide separate analysis for women who are both Black and Hispanic.

4 One should also note that this effect may be complicated by the effect of age; primiparous women (those who give birth for the first time) with higher levels of educational attainment are generally older than primiparous women with lower levels of education.

5 This response was translated from Spanish to English. The original response was: “que la atencios de los medicos fuera un poco mejo y no tubieran que esperar mucho, siun paciente dice me siento mal es porque de verdad se siete mal, no pruqe se quiera hacer tonta y la dejen esperando horas sentadas en la sala de espera.”
